# Acute paradoxical embolic cerebral ischemia secondary to possible May-Thurner syndrome and an atrial septal defect: a case report

**DOI:** 10.1186/1752-1947-7-172

**Published:** 2013-07-03

**Authors:** Richard A Rison, Matthew D Helfgott

**Affiliations:** 1 Los Angeles County Medical Center, PIH Health Stroke Program, University of Southern California Keck School of Medicine, 12401 Washington Blvd., Whittier, CA 90602, USA

## Abstract

**Introduction:**

May-Thurner syndrome is an anatomic abnormality that predisposes patients to increased risk of paradoxical embolism and stroke. It consists of chronic compression of the left common iliac vein by the overlying right common iliac artery which may predispose to local thrombus formation, which in turn may be the nidus of a paradoxical embolus leading to cerebral ischemia in patients with a right-to-left shunt secondary to an atrial septal defect or patent foramen ovale.

**Case presentation:**

We report the case of an embolic cerebral ischemic event in a 53-year-old Caucasian woman whose investigations revealed findings suggestive of possible May-Thurner syndrome coupled with an atrial septal defect. Her atrial septal defect was closed, she was placed on aspirin therapy, and has not had any recurrent events.

**Conclusion:**

May-Thurner syndrome is an important consideration in patients with paradoxical embolic cerebral ischemia and atrial septal defects.

## Introduction

May-Thurner syndrome (MTS) is an anatomic abnormality that has been associated with paradoxical embolism and stroke. In MTS, a deep vein thrombosis of the iliofemoral vein may form due to compression of the left common iliac vein by the overlying right common iliac artery, which can result in a paradoxical embolism if the patient also possesses a patent foramen ovale (PFO) or atrial septal defect (ASD) [1]. We report a case of an embolic cerebral ischemic event possibly resulting from MTS coupled with an ASD.

## Case presentation

A 53-year-old Caucasian woman presented to our Emergency Department with acute-onset speech impairment and coordination defects. She awoke in the morning to notice that when she took her dog for a walk that she had difficulty manipulating the leash and felt clumsy with her right hand. She also had difficulty with her memory in that she placed a water bowl down for her dog but forgot to place water in it. She felt disorganized with her thoughts and was concerned enough to call her brother, who noticed that her speech was slurred. Emergency medical services were activated and the patient was brought to PIH Health Hospital. She had no complaints of any headache, double vision, difficulty swallowing, vertigo, numbness, or focal weakness. There was no lapse of consciousness nor were there any drop attacks or history of seizures.

Her past medical history was significant for migraines with occasional visual auras and scoliosis. Family history was remarkable for an elderly grandmother who suffered a stroke. Personal habits included the use of occasional wine without history of tobacco or illicit drug use. Her medications at the time of admission included multivitamin supplements and hormone replacement therapy.

Physical examination revealed an afebrile, normotensive and pleasant woman who appeared her stated age and was in no acute distress. Neck examination did not reveal any carotid bruits and there were no murmurs noted on cardiac examination. Her cardiac rhythm was even.

Neurological examination demonstrated that she was awake and alert on mental status testing with intact recent and remote memory without any formal thought disorders. Speech examination showed some word-finding difficulty with word substitutions and mild stuttering. No dysarthria was noted but it was felt that she had a mild aphasia. Cranial nerves 2 to 12 were all intact without deficits. Motor examination revealed intact strength at 5 out of 5 on the Medical Research Council (MRC) scale throughout without drift, focal atrophy, or fasciculations. No abnormal involuntary movements were noted and tone was normal. Coordination testing revealed slight right-sided finger-to-nose dysmetria. Sensory testing demonstrated intact sensation to all primary sensory modalities with the exception of slight proprioceptive loss involving her right upper extremity. There was a mild loss of stereognosis of her right hand. Gait examination was unremarkable. Her reflexes were intact throughout without any long tract signs.

**Figure 1:**
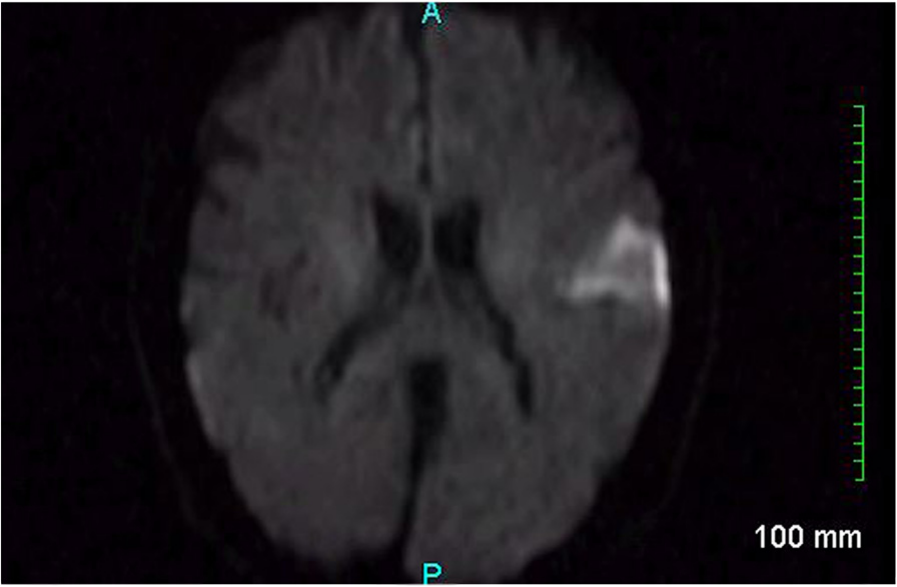
Diffusion-weighted magnetic resonance imaging demonstrating an acute ischemic infarct within the mid left temporal lobe.

Radiologic and sonographic investigations included an emergency head computed tomography (CT) scan, which was unremarkable. Subsequent magnetic resonance imaging (MRI) of the brain was performed and revealed an acute left parietal operculum ischemic infarct with mid left temporal lobe involvement (Figure 1) and findings suggestive of recent embolic infarcts in the centrum semiovale bilaterally. Carotid duplex imaging showed a less than 30% stenosis of the bilateral carotid arteries and transcranial Doppler studies were within normal limits. Magnetic resonance angiography (MRA) of the neck revealed a focal 2cm region of fusiform aneurysmal dilatation of the right subclavian artery along with a focal fusiform dilatation of the left brachiocephalic vein in the supraclavicular fossa with a diameter of approximately 1.1cm. MRA of the brain revealed a within normal limits intracranial circulation pattern. A chest X-ray did not reveal any acute cardiopulmonary disease.

Serologic studies included an elevated total cholesterol level of 218mg/dL. A complete blood count and metabolic panel were normal, and no coagulation defects were seen.

Given the brain MRI findings suggestive of a possible embolic pattern, both transthoracic and transesophageal echocardiography were performed, which revealed findings consistent with an ASD and a right-to-left shunt. Ejection fraction was normal at 70% without evidence of any pericardial effusion or intracardiac thrombus.

The findings of an ASD on echocardiography coupled with an embolic pattern seen on the brain MRI scan lead to a magnetic resonance venogram (MRV) of the pelvis. The MRV revealed effacement of the proximal left common iliac vein due to impression by the adjacent left iliac common iliac artery without evidence of thrombosis, which was felt to be consistent with MTS.

The patient was placed on aspirin 325mg daily and asked to stop her hormone replacement therapy. The vascular surgery department was consulted and did not recommend any surgical intervention regarding her MTS or for her right subclavian fusiform aneurysmal dilatation or her focal fusiform dilatation of the left brachiocephalic vein. She was subsequently referred to a local tertiary facility where she underwent closure of her ASD.

The patient has been doing quite well to date with five years of follow-up without any new cerebral ischemic events. She remains active and functionally independent.

## Discussion

Cerebral emboli have been reported to account for approximately 15% of all ischemic strokes [1]. ASDs and PFOs are congenital cardiac defects that have been associated with paradoxical cryptogenic ischemic stroke. ASDs are detected in one child per 1500 live births, and make up 30 to 40% of all congenital heart diseases that are seen in adults. PFOs are quite common (appearing in 10 to 20% of adults) [2]. Often however, the actual site of the origin of the embolus goes undetected despite routine investigations including standard echocardiography, even when an ASD or PFO is seen. In these situations including the absence of cardiac dysrhythmias, further investigations are warranted.

MTS has been reported to be a possible cause of paradoxical emboli, which can go undetected if the patient is screened for venous thrombosis by lower-extremity venous Doppler alone. MTS is caused by a mechanical obstruction of the common iliac vein and is associated with recurrent deep vein thrombosis. The first description of MTS was in 1956 and termed ‘iliac compression syndrome’ [3]. MTS consists of venous flow impedance of the left common iliac vein by the overlying right common iliac artery, which may lead to intimal hypertrophy, further flow impedance, and thrombus formation secondary to chronic pulsatile forces. MTS has been estimated to occur in 20% of the population and the average age of onset is between 18 and 30 years of age and is approximately five times more common in women than men [1,3-6].

In our patient’s case, although the brain CT was negative for any acute cerebral ischemia findings (not unusual within the acute period of an ischemic stroke), brain MRI studies were suggestive of an embolic pattern with predominant left-sided parietal and mid-left temporal lobe involvement (as demonstrated by her speech difficulties). The neck MRA findings were felt to be incidental by our team and the vascular surgery department and no surgical intervention was recommended. Further investigations revealed an ASD and subsequently MTS seen on MRV. Although contrast venography has traditionally been the gold standard diagnosis of MTS, the findings can also be seen on modern MRV. Surgery is often advised since conservative therapy such as with systemic anticoagulation is often not effective [5,6], as recurrences of deep venous thrombosis and related symptoms are common [1,5,6]. However in our patient’s case, she never had a documented lower extremity deep vein thrombosis and hence the vascular surgery department recommended only conservative therapy with observation.

We hypothesize that our patient had a lower extremity venous thrombus caused by MTS that may have caused her paradoxical embolic cerebral ischemic event via her ASD. We did not see any venous thrombus on our investigations, probably because it had already embolized. Although a direct correlative cause from her MTS is lacking, our patient underwent ASD closure, and to date has not had any recurrent cerebral ischemic events on daily aspirin and no physical examination findings suggestive of lower extremity deep vein thrombosis with five years of follow-up.

## Conclusion

MTS may be a cause of paradoxical cerebral emboli, especially in patients with an ASD. All clinicians treating stroke patients should be aware of MTS.

## Consent

Written informed consent was obtained from the patient for publication of this case report and any accompanying images. A copy of the written consent is available for review by the Editor-in-Chief of this journal.

## Competing interests

The authors declare that they have no competing interests.

## Authors’ contributions

RAR examined the patient. MDH and RAR analyzed and interpreted the patient data. MDH provided the initial draft of the manuscript. RAR revised and edited the manuscript. Both authors read and approved the final manuscript.

## Authors’ information

RAR is a Deputy Editor for the *Journal of Medical Case Reports* and is an Associate Neurology Editor for *Grand Rounds* and *WebmedCentral*. RAR also serves as a Section Editor for *BMC Research Notes*. RAR practices general neurology at Neurology Consultants Medical Group, serves as Medical Director of the PIH Health Stroke Program, is a Clinical Assistant Professor of Neurology at the University of Southern California-Keck School of Medicine-Los Angeles County Medical Center, and is a Fellow of the American Association of Neuromuscular and Electrodiagnostic Medicine. RAR is board certified by the American Board of Psychiatry and Neurology in Neurology and Vascular Neurology, and is board certified by the United Council for Neurologic Subspecialties in Neurocritical Care and Neuroimaging. RAR is also board certified by the American Board of Electrodiagnostic Medicine in Electrodiagnostic Medicine and is a former president of the Los Angeles Neurological Society. MDH received a B.A. degree from the University of Southern California and just completed Scripps College Post-Baccalaureate Premedical Program.

## References

[B1] Way J Lopez-Yunez A Beristain X Biller J Paradoxical embolism to the basilar apex associated with May-Thurner syndrome Arch Neurol 2000 57 1761 1764 10.1001/archneur.57.12.1761 11115242

[B2] Kaplan S Congenital heart disease in adolescents and adults. Natural and postoperative history across age groups Cardiol Clin 1993 11 543 556 8252558

[B3] May R Thurner J The cause of the predominantly sinistral occurrence of thrombosis of the pelvic veins Angiology 1957 8 419 427 10.1177/000331975700800505 13478912

[B4] Greer DM Buonanno FS Cerebral infarction in conjunction with patent foramen ovale and May-Thurner syndrome J Neuroimaging 2001 11 432 434 10.1111/j.1552-6569.2001.tb00074.x 11677885

[B5] Peters M Syed RK Katz M Moscona J Press C Nijjar V Bisharat M Baldwin D May-Thurner syndrome: a not so uncommon cause of a common condition Proc (Bayl Univ Med Cent) 2012 25 231 233 10.1080/08998280.2012.11928834 PMC3377287 22754121

[B6] Kiernan TJ Yan BP Cubeddu RJ Rengifo-Moreno P Gupta V Inglessis I Ning M Demirjian ZN Jaff MR Buonanno FS Schainfeld RM Palacios IF May-Thurner syndrome in patients with cryptogenic stroke and patent foramen ovale: an important clinical association Stroke 2009 40 1502 1504 10.1161/STROKEAHA.108.527366 19182088 PMC3744111

